# Vasoconstrictive responses by the carotid and auricular arteries in goats to ergot alkaloid exposure^1^

**DOI:** 10.3389/fchem.2014.00101

**Published:** 2014-11-20

**Authors:** Glen E. Aiken, Michael D. Flythe

**Affiliations:** Forage-Animal Production Research Unit, United States Department of Agriculture Agricultural Research ServiceLexington, KY, USA

**Keywords:** ergot alkaloids, fescue toxicosis, goats, tall fescue, vasoconstriction

## Abstract

A fungal endophyte (*Neotyphodium coenophialum*) infects most plants of “Kentucky 31” tall fescue (*Lolium arundinaceum*) and produces ergot alkaloids that cause persistent constriction of the vascular system in grazing livestock. Consequently, animals undergoing this toxicosis cannot regulate core body temperature and are vulnerable to heat and cold stresses. An experiment was conducted to determine if the caudal and auricular arteries in goats (*Capra aegagrus hircus*) vasoconstrict in response to ergot alkaloids. Seven, rumen fistulated goats were fed *ad libitum* orchardgrass (*Dactylis glomeratia*) hay and ruminally infused with endophtye-free seed (E−) for a 7-day adjustment period. Two periods followed with E− and endophyte-infected (E+) seed being randomly assigned to the 2 goat groups in period 1 and then switching treatments between groups in period 2. Infused E+ and E− seed were in equal proportions to the hay such that concentrations of ergovaline and ergovalanine were 0.80 μg per g dry matter for the E+ treatment. Cross-sections of both arteries were imaged using Doppler ultrasonography on days 0, 2, 4, 6, 8, and 12 in period 1 and on days 0, 1, 2, 3, 6, 7, and 9 in period 2. Differences from average baseline areas were used to determine presence or absence of alkaloid-induced vasoconstriction. Carotid arteries initiated constriction on imaging day 2 in both periods, and auricular arteries initiated constriction on imaging day 2 in period 1 and on day 6 in period 2. Luminal areas of the carotid arteries in E+ goats were 46% less than baseline areas in both periods after vasoconstriction occurred, whereas auricular arteries in E+ goats were 52% less than baseline areas in period 1 and 38% in period 2. Both arteries in E+ goats in period 1 relaxed relative to baseline areas by imaging day 2 after they were switched to the E− treatment. Results indicated that goats can vasoconstrict when exposed to ergot alkaloids that could disrupt their thermoregulation.

## Introduction

Tall fescue is a cool-season, perennial grass that is extensively utilized for grazing and hay production on approximately 15 million hectares in the eastern half of the USA. The grass is persistent and productive under low management, but this benefit is primarily due to alkaloids produced by a *Neotyphodium* endophyte (Hoveland et al., 1983; Strickland et al., 2011) that inhabits the intracellular spaces of most tall fescue plants. Groups of alkaloids (e.g., lolines and peramines) are recognized for providing the plant with tolerances to environmental stresses, such as drought and herbivory (Siegel et al., 1990; Bacon, 1993).

Ergot alkaloids are a class of alkaloids also produced by the endophyte that causes a toxicosis in grazing livestock that reduces both reproductive performance (Porter and Thompson, 1992) and growth rate (Schmidt and Osborn, 1993; Strickland et al., 1993; Paterson et al., 1995). Ergot alkaloids bind biogenic amine receptors in the vasculature to induce persistent vasoconstriction (Oliver, 2005). Constricted blood flow to peripheral tissues reduces the animal's ability to regulate body temperature and, therefore, be vulnerable to heat and cold stress (Aldrich et al., 1993). Ergovaline is an ergopeptine ergot alkaloid that was demonstrated to have the highest vasoconstrictive potency of the ergot alkaloids (Klotz et al., 2008) and also is of highest concentration, ranging from 84 to 97% of the ergopeptine fraction (Lyons et al., 1986).

Browning (2012) reported reduced growth rates of Boer, Kiko, and Spanish goats *(Capra aegagrus hircus)* fed diets containing 1.16 ppm ergovaline as compared to a diet free of ergot alkaloids. However, alterations in the physiology of goats grazing toxic endophyte-infected tall fescue have not been investigated (Ditsch and Aiken, 2009), but is needed as the goat industry expands into the transition zone and goats are exposed to toxic ergot alkaloids. Vasoconstrictive responses to ergot alkaloids have been reported in cattle (Rhodes et al., 1991; Aiken et al., 2009b), sheep (Aiken et al., 2011), and horses (McDowell et al., 2013), but not in goats. Therefore, a pen study was conducted using Color Doppler ultrasonography to compare luminal areas of carotid and auricular arteries in wether goats that were fed either endophyte-infected (E+) or endophyte-free (E−) tall fescue seed.

## Materials and methods

The experiment was conducted in indoor pens under a controlled ambient temperature of approximately 20°C. All animal research followed procedures approved by the University of Kentucky Institutional Animal Care and Use Committee (protocol number 2013-1152).

Seven Spanish wether goats that were ruminally fistulated (age = yearling; body weight = 34.3 ± 1.7 kg) were randomly assigned to 4 goats in one pen group and 3 in the other group to compare luminal areas of the carotid and auricular arteries between diets containing either E+ or E− tall fescue seed. A 7-d adjustment period was conducted to measure DM intake of commercially chopped orchardgrass (*Dactylis glomerata* L.) hay. Two experimental periods followed the adjustment period as a crossover design, with the group of 3 goats receiving the E+ seed in the first period and with the treatments being switched between the two groups at the beginning of period 2. Rumens were infused daily at an average of 143 and 142 g of E− or E+ seed, respectively, during period 1 and 135 and 170 g of E− or E+ seed, respectively, during period 2. Duration of period 1 was 14 days and for period 2 was 13 days.

The cultivar “Defiance” was used for the E+ treatment and the cultivar “Kentucky 32” was used for the E− treatment. Ergovaline plus its epimer, ergovalanine were analyzed for both cultivars using procedures of Yates and Powell (1998) and modified as described by Carter et al. (2010).

Hay was fed *ad libitum* and seed that was previously course ground using a Wiley Mill with a 5.0 mm screen was infused into each rumen using a funnel. Feeding of hay and treatment with seed was done at 1500 h each day. During the experimental periods the previous day's intake for each pen were used to estimate intake per goat and the ratio of fed chopped hay to rumenally placed seed for providing a daily diet concentration of 0.8 μg of ergovaline and ergovalanine, per gram of dry matter. Ergovaline plus ergovalanine concentrations in E+ and E− seed contained 2.71 and 0 μg per gm DM, respectively, which resulted in seed averaging 14.5% of the diet dry matter for the E+ and E− treatments.

**Figure 1:**
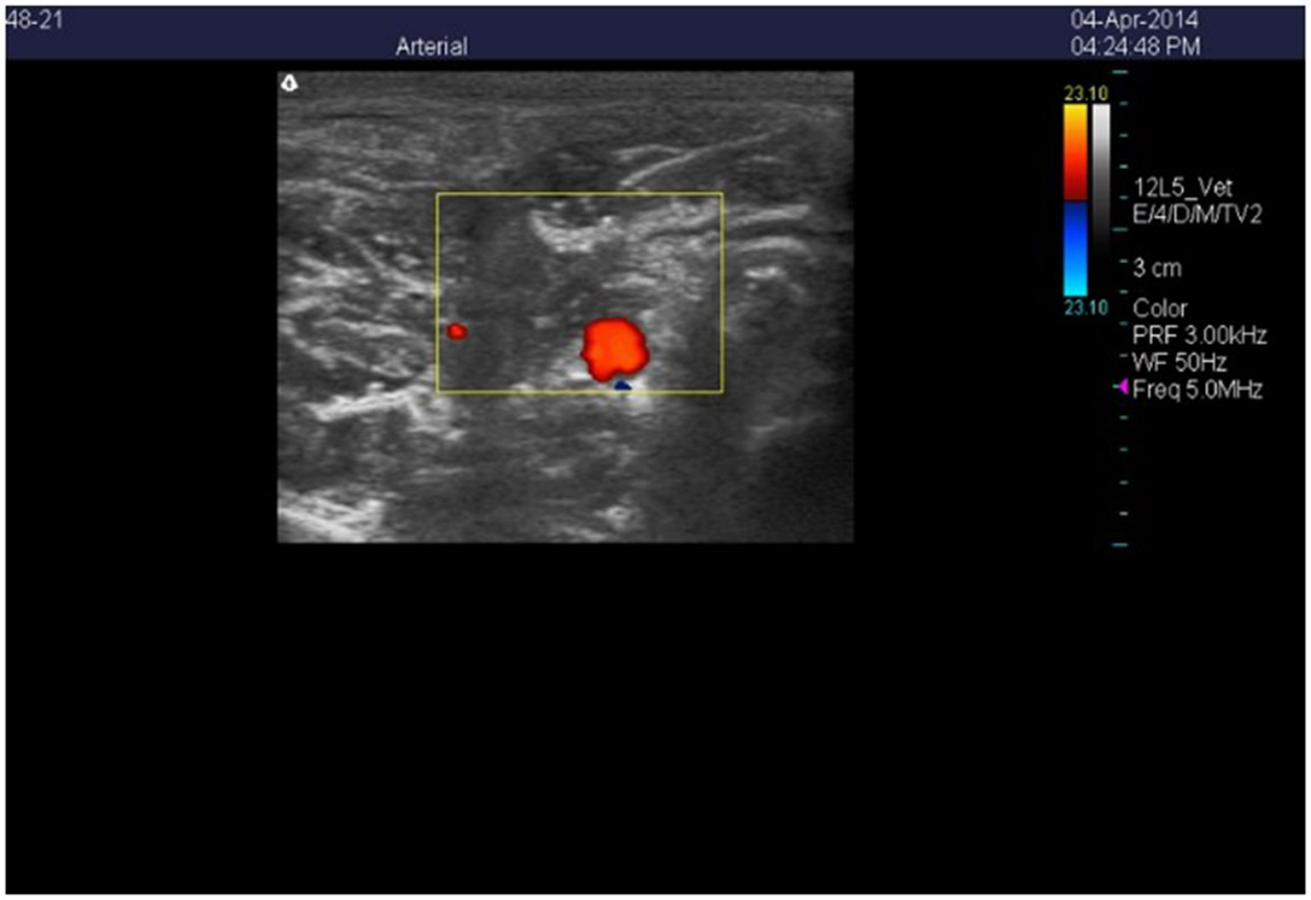
**Color Doppler ultrasonic image of the cross-section of the right carotid artery in a wether goat on an orchardgrass diet with no ruminal infusion of endophyte-infected or endophyte-free tall fescue seed**. Color delineates blood flow in the lumen of the artery.

**Figure 2:**
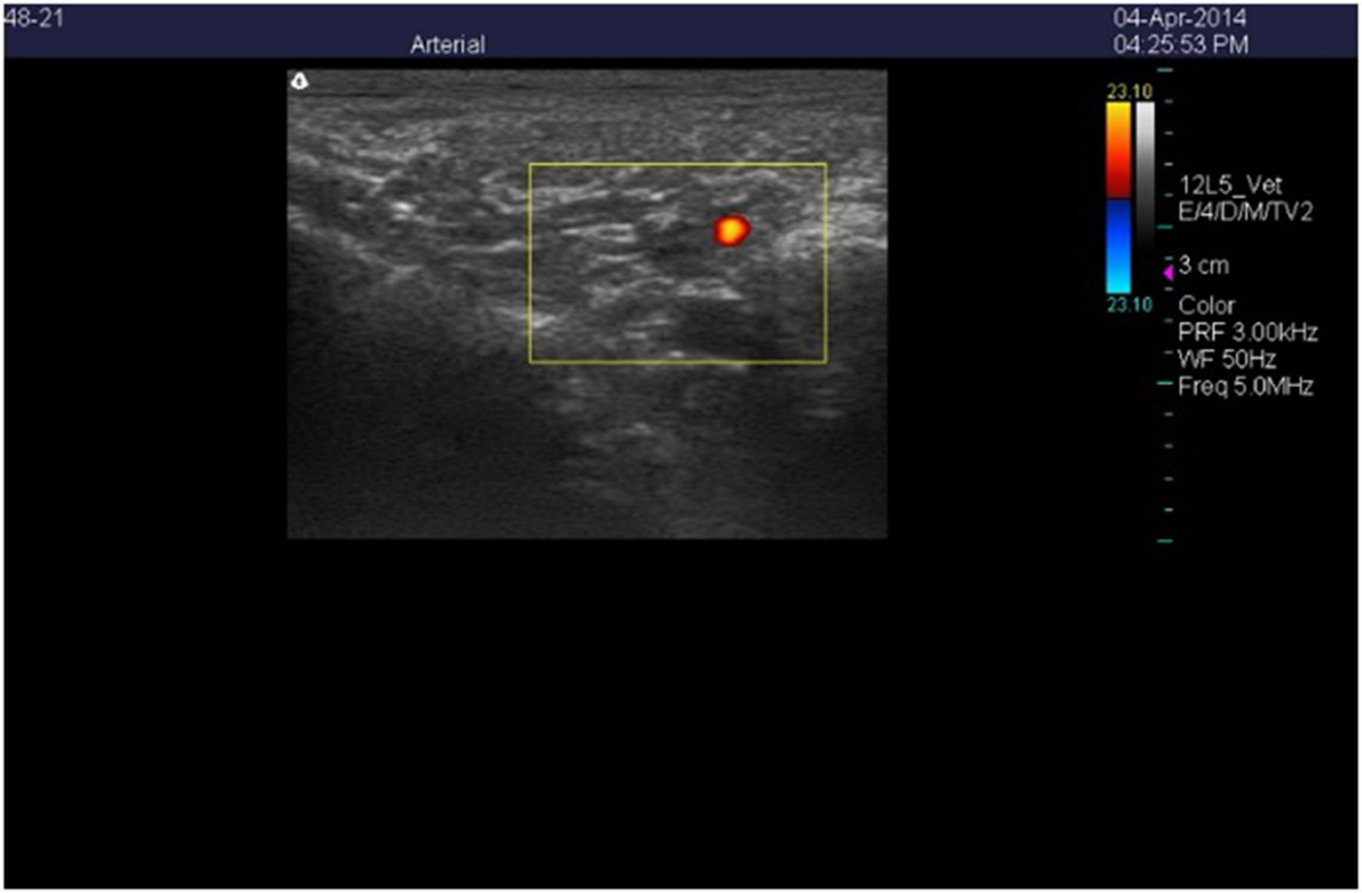
**Color Doppler ultrasonic image of the cross-section of the right auricular artery in a wether goat on an orchardgrass diet with no ruminal infusion of endophyte-infected or endophyte-free tall fescue seed**. Color delineates blood flow in the lumen of the artery.

Color Doppler ultrasound images of the cross-sections of the right carotid (Figure 1) and auricular (Figure 2) arteries were collected using a Classic Medical TeraVet 3000 Ultrasound Unit (Classic Universal Ultrasound, Tequesta, FL) with a 12L5-VET (12 MHz) linear array transducer. Baseline measures were collected for each goat during the adjustment period on days 3, 5, and 7. Images were collected during period 1 on days 1, 2, 4, 6, 8, and 14, and were collected during period 2 on days 1, 2, 3, 4, 7, 8, and 10. Each imaging session was started at approximately 1100 h and was completed within 30 to 40 min. The goats were handled frequently prior to the start of the study to reduce excitability when being imaged. Individual goats were removed from their pens and their heads were stabilized by gently holding their horns without being placed in a chute. Each goat was clipped at the start of the experiment using surgical clippers under the left ear cross-sectional images were collected for each artery using a frequency of 5.0 MHz and a pulse repetitive frequency that ranged between 2.5 and 3.0 kHz. Scan depth was set at 4 cm for the carotid arteries and 3 cm for the auricular artery. Following freezing of an individual scan, frames stored in the cine memory of the unit were searched to store the image exhibiting the maximum flow signal and assumed to be at peak systolic phase. The flow signal was traced to estimate lumen area (Aiken et al., 2009a).

Caudal and auricular artery luminal areas were analyzed using mixed models of SAS as repeated measures with the heterogeneous autoregressive covariance structure for responses of carotid arteries in periods 1 and 2 and auricular arteries in period 2 and with the autoregressive covariance structure for the auricular arteries in period 1 (Littell et al., 1996). The analysis used individual animals as the experimental unit. Treatment (E+ vs. E−), imaging day, and the interaction between treatment and imaging day were analyzed as fixed effects for both experiments. Measures for the adjustment period were averaged and used as the baseline measure (image day 0) for period 1 and those for period 1 were averaged for each treatment and used as the baseline measure for period 2. In the presence of a significant (*P* < 0.05) treatment x imaging day interaction, differences in least square means between baseline measures and imaging days and differences between treatments at each imaging day were determined using the PDIFF option of SAS.

## Results

Mean dry matter intake during the adjustment period was 0.97 kg/goat. During period 1, it was 0.80 and 0.81 kg/goat for E− and E+ treatments, respectively, and during period 2 it was 0.76 and 0.97 kg/goat, respectively. Consumption of ergovaline and ergovalanine averaged approximately 1.1 × 10^−5^ μg per kg body weight.

### Period 1

**Figure 3:**
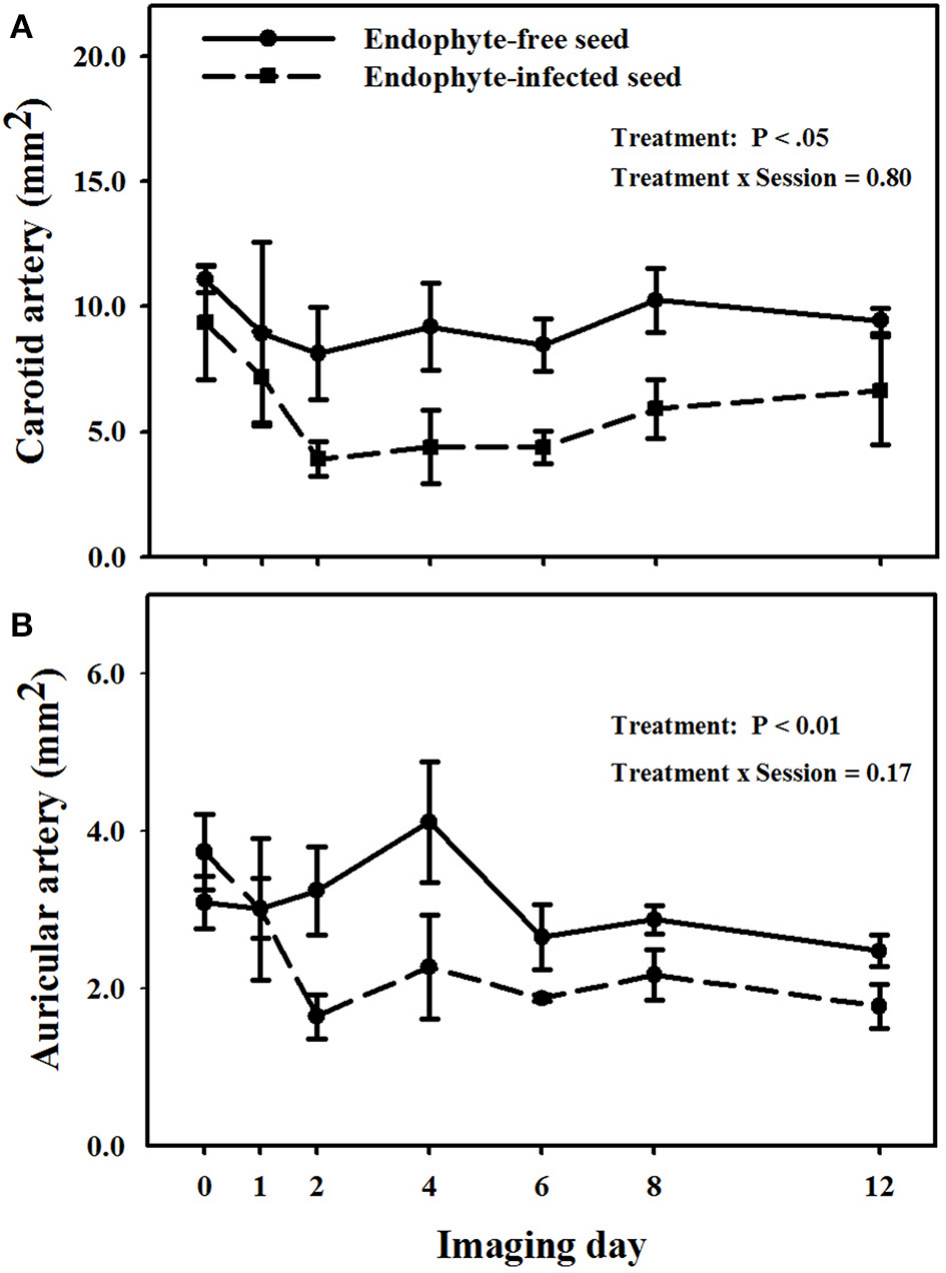
**Trends over image days during period 1 in luminal area of the right (A) carotid and (B) auricular arteries in weither goats that were ruminally infused with either endophyte-infected or endophyte-free tall fescue seed**. The day 0 is the average of luminal areas during the adjustment period. Standard errors of the mean were calculated using the conservative formula.

Mean luminal areas of the carotid arteries in E+ goats were less (*P* < 0.05) than those in E− goats, and there was no treatment × imaging day interaction (*P* = 0.805; Figure 3A). Mean luminal area for goats on the E− treatment during the period was 9.4 ± 0.9 mm^2^ and for those on the E+ treatment it was 5.8 ± 1.1 mm^2^.

Different from carotid arteries, there was a treatment × imaging day interaction (*P* < 0.001) on luminal areas of the auricular arteries (Figure 3B). Luminal areas of auricular arteries for the E− treatment were not different (*P* > 0.12) from the mean baseline value, whereas those for the E+ treatment differed from mean baseline areas for all imaging days on and after day 2. Auricular arteries in E+ goats showed vasoconstriction (*P* < 0.05) as compared to E− goats on the imaging sessions for days 2, 4, and 12, even though after the second imaging day lumen areas in E+ goats were never greater than 2.3 mm^2^ and in E− goats they were never less than 2.5 mm^2^.

### Period 2

**Figure 4:**
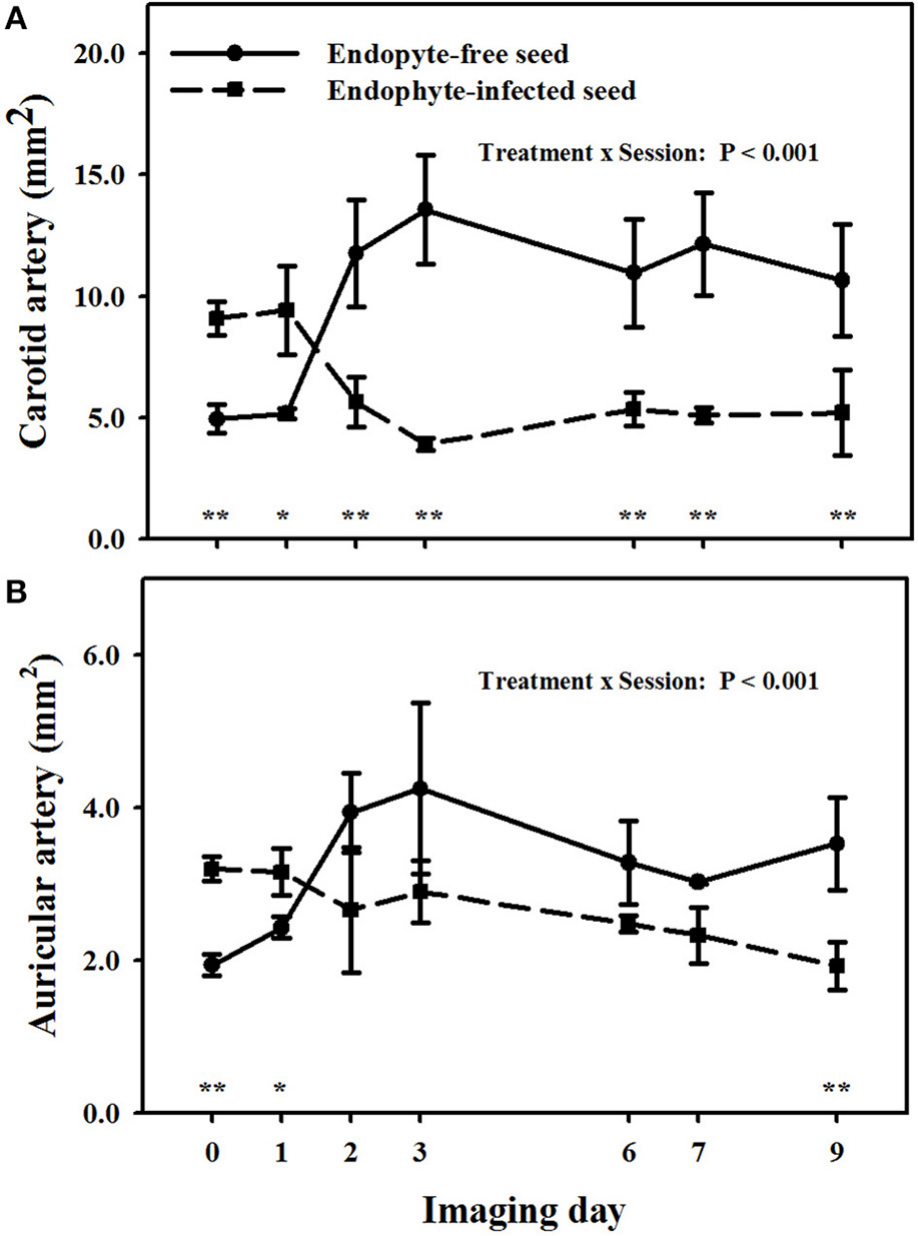
**Trends over image days during period 2 in luminal area of the right (A) carotid and (B) auricular arteries in weither goats that were ruminally infused with either endophyte-infected or endophyte-free tall fescue seed**. The day 0 is the average of luminal areas during period 1 before treatments were switched. Standard errors of the mean were calculated using the conservative formula. Differences between treatments for imaging days and denoted by ^*^P < 0.10 and ^**^P < 0.05.

There was a treatment × imaging day interaction (*P* < 0.001) on carotid arteries (Figure 4A). Goats that were switched to E+ exposure after being on the E− treatment in period 1 showed less luminal areas than the baseline measure on and after imaging day 3. Luminal areas for the E+ treatment were less (*P* < 0.05) than those for the E− for imaging days 2, 3, 6, and 7, whereas there was a tendency (*P* < 0.10) for a difference on the day 9 imaging when the standard errors for both treatment groups were highest.

A treatment × imaging day interaction also was detected (*P* < 0.01) for luminal areas of auricular arteries (Figure 4B). Auricular arteries in goats switched to E− from the E+ treatment in period 1 were relaxed from the baseline measure on and after day 2. Those in goats switched to E+ in period 2 had a tendency (*P* < 0.10) for vasoconstriction as compared to baseline measures on day 2, and exhibited significant (*P* < 0.05) vasoconstriction on and after day 3. Luminal areas in the E+ goats tended (*P* < 0.10) to be less (*P* ≤ 0.05) than for the E− goats day 1, but exhibited significant (*P* < 0.05) vasoconstriction on and after day 2 (*P* < 0.05).

## Discussion

Blood flow resistance under normal conditions is regulated by vasoconstriction and vasorelaxation/vasodialation of arteries and veins that is controlled by the autonomic nervous system. Blood flow volume is proportional to the fourth power of luminal radius, which results in large decreases in blood flow volume occurring with small decreases in luminal area (Carter, 2000). Therefore, responses of luminal areas of blood vessel cross-sections provide a direct measure of vasoconstriction or vasorelaxation that has a direct bearing on blood flow volume.

Although ergovaline is the ergopeptine of highest concentration and has the highest potency, other ergot alkaloids were assumed to be in the seed that could have additive effects on vasoconstriction (Klotz et al., 2010). Klotz et al. (2007) used an *in vitro* procedure with lateral saphenous veins biopsied from endophyte-naïve heifers to determine that ergovaline elicited a vasoconstrictive response at a concentration 1 × 10^−8^ M and had a maximum contractility of 69.9% relative to norepinephrine contractility. From an earlier experiment, Klotz et al. (2006) reported a concentration greater than 1 × 10^−4^ M was necessary for lysergic acid to induce vasoconstriction with a maximum contractility of 15.6% relative to norepinephrine contractility. Klotz et al. (2008) later reported that ergonovine, ergocryptine, ergocristine, and ergocornine induced a contractile response at similar concentrations (1 × 10^−7^ M) to ergovaline, but the greatest maximum contractility intensity relative to norepinephrine was achieved by ergonovine (68.5%), and maximum contractility intensities were similar between ergocryptine (45.5%), ergocristine (42.9%), and ergocornine (57.2%). Although ergovaline has demonstrated to be the most potent vasoconstrictor, Klotz et al. (2009) surmised that ergopeptines likely have additive effects on intensity of vascular contraction, thus indicating all are likely contributors to fescue toxicosis.

Overall, luminal areas tended to be more variable in E− goats than in E+ goats. This was clearly indicated by the responses of carotid arteries in both periods and the auricular arteries in period 2 which had Akaike's Information Criterion values in statistical analyses that favored the heterogeneous autoregressive covariance structure, which accounts for heterogeneity of treatment variances (Littell et al., 1996). Luminal areas of caudal arteries in cattle also have been observed to be more variable with E− than E+ diets (Aiken et al., 2007, 2009a). Alkaloid-induced vasoconstriction apparently reduces vascular responsiveness to environmental stimuli.

The goats used in the study were not endophyte-naïve. Prior to placement in the indoor pens they resided in a mixed grass pasture that had moderate percentages of endophyte-infected tall fescue; however, they had been on a non-toxic hay diet in the pens for 38 days prior to the 7 day adjustment period when their rumens were daily infused with E− seed. It was assumed that most of the ergot alkaloids had cleared from the vasculature prior to initiating the E+ diet, which was indicated by the alkaloid-induced vasoconstriction that occurred in both arteries during period 1. Overall luminal areas of carotid and auricular arteries were less (*P* < 0.05) than baseline measures taken during the adjustment periods by day 2.

Day-to-day fluctuations in luminal areas in both arteries in E+ goats that were parallel with those in E− goats are indicative of these goats not being saturated with ergot alkaloids and still having an ability to make vascular adjustments. Using an *in vitro* model with biopsied saphenous veins from endophyte-naïve heifers, Klotz et al. (2009) reported increased smooth muscle contractility with increasing repetitive additions of 1 × 10^−7^ M concentrations of ergovaline. It was further shown that ergovaline bioaccumulated in the saphenous veins with the repeated exposures and washings. Therefore, this indicates that ergovaline can bioaccumulate in the bovine vasculature through their affinity to biogenic amine receptors.

Switching treatments between the two goat groups in period 2 allowed for an evaluation of possible recovery of the vascular system to previous ergot alkaloid exposure. Detection of relaxation by the carotid and auricular arteries starting on image day 2 indicated that the goats were not saturated with ergot alkaloids by completion of feeding E+ seed during period 1. Aiken et al. (2011) reported luminal areas of auricular arteries in ewe lambs to linearly increase over time after they were switched to E− perennial ryegrass pasture after 19 days of grazing E+ pasture.

Vasoconstrictive responses in period 2, as detected by differences with baseline measures during period 1, were not as immediate as in period 1, with significant differences from baseline measures from period 1 not being observed until image day 3 for the carotid artery and day 6 for the auricular arteries. Treatment differences on individual imaging days cannot be interpreted because of confounding between relaxation of the arteries in goats that were switched from E+ to E− diets and vasoconstriction in those that were switched from E− to E+ diets. Nonetheless, the objectives of period 2 were to evaluate differences in luminal areas of the arteries between period 2 images and baselines means for detecting artery relaxation in E− goats and alkaloid-induced vasoconstriction in E+ goats.

## Conclusion

Results of this experiment indicated the vasculature of goats can vasoconstrict when exposed to ergot alkaloids. Alkaloid-induced vasoconstriction of the carotid and auricular arteries was mediated within 1 to 2 days after being exposed to ergot alkaloids during period 1, and 2 to 3 days during period 2. Luminal areas of carotid and auricular artereries in E+ goats during period 1 showed rapid increases after they were switched to the E− in period 2, with significant relaxation being accomplished in 2 days for both arteries. The 12-day exposure to ergot alkaloids was short and likely was not long enough for vascular systems in the goats to be saturated with alkaloids. This experiment provides the first documentation of ergot alkaloid-induced vasoconstriction in goats.

### Conflict of interest statement

The Reviewer Joan M. Burke declares that, despite being affiliated to the same institution as authors Glen E. Aiken and Michael D. Flythe, the review process was handled objectively and not conflict of interest exists. The authors declare that the research was conducted in the absence of any commercial or financial relationships that could be construed as a potential conflict of interest.
